# A Randomized Comparison of the Healing Response Between the Firehawk Stent and the Xience Stent in Patients With ST-Segment Elevation Myocardial Infarction at 6 Months of Follow-Up (TARGET STEMI OCT China Trial): An Optical Coherence Tomography Study

**DOI:** 10.3389/fcvm.2022.895167

**Published:** 2022-06-01

**Authors:** Yuan He, Rutao Wang, Jianzheng Liu, Fei Li, Jiayi Li, Chengxiang Li, Jingyu Zhou, Zhijing Zhao, Wangwei Yang, Fangjun Mou, Jing Wang, Jing Kan, Xiaobo Li, Yan Li, Ming Zheng, Shaoliang Chen, Chao Gao, Ling Tao

**Affiliations:** ^1^Department of Cardiology, Xijing Hospital, Air Force Medical University, Xi'an, China; ^2^Department of Cardiology, Radboud University, Nijmegen, Netherlands; ^3^Department of Cardiology, National University of Ireland, Galway, Ireland; ^4^Department of Cardiology, Nanjing First Hospital, Nanjing Medical University, Nanjing, China; ^5^Department of Cardiology, Tangdu Hospital, Air Force Medical University, Xi'an, China; ^6^Shanghai MicroPort Medical (Group), Shanghai, China

**Keywords:** biodegradable polymer, neointimal thickness, optical coherence tomography, ST-segment elevation myocardial infarction, durable polymer

## Abstract

**Background:**

The healing response of the Firehawk stent in patients with ST-segment elevation myocardial infarction (STEMI) remains unclear.

**Aim:**

We compared the vascular healing of a biodegradable polymer sirolimus-eluting stent (Firehawk) vs. a durable polymer everolimus-eluting stent (Xience) at 6 months after percutaneous coronary intervention (PCI) in patients with STEMI.

**Methods:**

In this prospective, multicenter, randomized, non-inferiority study, patients within 12 h of STEMI onset were randomized in a ratio of 1:1 to receive Firehawk or Xience stents. Optical coherence tomography (OCT) follow-up was performed 6 months after the index procedure and assessed frame by frame. The primary endpoint was the neointimal thickness (NIT) at 6 months evaluated by OCT. The safety endpoint was target lesion failure (TLF) at 12 months.

**Results:**

The Firehawk stent was non-inferior to the Xience stent in terms of the neointimal thickness (73.03 ± 33.30 μm vs. 78.96 ± 33.29 μm; absolute difference: −5.94 [one-sided 95% lower confidence bound: −23.09]; *P*_non−inferiority_ < 0.001). No significant difference was observed between the Firehawk and Xience groups regarding the percentage of uncovered struts (0.55 [0.08, 1.32]% vs. 0.40 [0.21, 1.19]%, *P* = 0.804), the percentage of malapposed struts (0.17 [0.00, 1.52]% vs. 0.17 [0.00, 0.69]%, *P* = 0.662), and the healing score (1.56 [0.23, 5.74] vs. 2.12 [0.91, 3.81], *P* = 0.647). At 12 months, one patient in the Firehawk group experienced a clinically indicated target lesion revascularization. No other TLF events occurred in both groups. Independent risk factors of the NIT included body mass index, hyperlipidemia, B2/C lesions, thrombus G3–G5, thrombus aspiration, and postdilation pressure.

**Conclusion:**

In patients with STEMI, Firehawk was non-inferior to Xience in vascular healing at 6 months. Both stents exhibited nearly complete strut coverage, moderate neointimal formation, and minimal strut malapposition.

**Clinical Trial Number:**

NCT04150016.

## Introduction

Delayed vascular healing or incomplete endothelialization is related to late or very late stent thrombosis (ST) (1, 2). Studies have found that patients with ST-segment elevation myocardial infarction (STEMI) show a higher incidence of ST than patients with stable coronary artery disease (CAD) (3, 4). Pathological and optical coherence tomography (OCT) studies have documented greater inflammation and more frequent incomplete endothelialization in patients with STEMI than patients with stable CAD after drug-eluting stent (DES) implantation (5–8). Previous studies have explored the risk factors of ST and have showed that incomplete stent apposition, type of stent (bare-metal stent, first-generation DES, second-generation DES), interruption of dual antiplatelet therapy (DAPT), history of myocardial infarction or percutaneous coronary intervention (PCI), current smoking, insulin-treated diabetes mellitus, and platelet activity are associated with late or very late ST in patients with STEMI (8–11). The prothrombotic and inflammatory milieu in STEMI poses particular challenges to vascular healing and stent-related clinical outcomes after primary PCI (5, 12, 13).

The current device technologies may offer improved outcomes in patients with STEMI. Biodegradable-polymer or polymer-free DESs were developed with the purpose of controlling drug release, allowing the dissolution of the polymer material simultaneously or subsequently to eliminate the stimulus for chronic inflammation and improve stent safety (14–17). Although antiproliferative drug coatings in stents are vital to reduce restenosis, they can delay vascular healing and have been linked to late ST (18, 19). The placement of a DES with a lower but still effective drug dose and more optimized release curves has led to advantages in vascular healing, therefore, improving clinical outcomes (20–22).

The Firehawk sirolimus-eluting stent (MicroPort Medical, Shanghai, China) is a thin-strut (86 μm) cobalt–chromium stent platform with a fully biodegradable polylactic acid polymer coating applied to abluminal grooves to minimize polymer burden and reduce drug concentrations (sirolimus concentration, 0.3 μg/mm^2^). The design of the Firehawk stent is intended to balance anti-restenosis and vascular healing by minimizing the inflammatory response (23). In the TARGET All Comers trial, the Firehawk stent was proven to be non-inferior to the Xience stent (a durable polymer everolimus-eluting stent; Abbott Vascular, Santa Clara, CA, USA) for target lesion failure (TLF) at 12 months (23). The OCT substudy of TARGET All Comers demonstrated that the Firehawk and Xience stents have comparable vascular healing profiles at 3 months after stent implantation in patients presenting with stable CAD or acute coronary syndrome (24); however, patients with STEMI were excluded from this study, and the vascular response in patients with STEMI with Firehawk stents remains unknown.

In this study (TARGET STEMI OCT China trial), we compared the OCT-assessed neointimal thickness (NIT) of the Firehawk and Xience stents at 6 months after primary PCI in a STEMI population.

## Methods

### Study Design

This study (TARGET STEMI OCT China trial, NCT04150016) was a prospective, multicenter, randomized, noninferiority trial comparing the Firehawk abluminal groove-filled biodegradable polymer sirolimus-eluting stent and the Xience durable polymer everolimus-eluting stent (1:1 allocation). The randomization procedure was implemented by a sealed envelope method and stratified according to the center. Primary PCI was performed according to the current guidelines (25). Pre- and post-stenting dilation, thrombus aspiration, and complete revascularization were performed according to the operators' discretion. Loading doses of the P2Y_12_ inhibitor (clopidogrel 600 mg or ticagrelor 180 mg) and aspirin (300 mg) were prescribed to patients before the procedure if they did not take aspirin and a P2Y_12_ inhibitor routinely. Patients were discharged with a prescription of aspirin 100 mg indefinitely and clopidogrel 75 mg once per day or ticagrelor 90 mg twice per day for 12 months after the index procedure.

The study protocol was approved by the Ethics Committees of all participating sites, and the study was performed in accordance with local laws and regulations and complied with the Declaration of Helsinki. Written informed consent was obtained from patients before the randomization.

### Study Device

The Firehawk stent is a balloon-expandable L605 cobalt–chromium stent platform with a strut thickness of 86 μm and a cell area of 4.73 mm^2^ for the 3.5-mm stent. The polymer, with a 10-μm thickness, is a D,L-polylactic acid biodegradable polymer and embedded in grooves on the abluminal strut surface, which provides controlled release of the antiproliferative drug. The antiproliferative sirolimus drug density is 0.3 μg/mm^2^, with 90% release by 90 days. The polymer biodegrades within 6–9 months, leaving only the metallic stent as a permanent implant.

The Xience is a laser-cut cobalt–chromium stent with an 81-μm strut thickness coated with a 7.7-μm durable fluoride-hexafluoropropylene polymer. The everolimus drug density is 1 μg/mm^2^, with a full release in 120 days.

### Study Population

Details of the eligibility criteria of the current study are presented in the Supplementary Material. Briefly, we enrolled patients who met all the following criteria: (1) consecutive patients aged ≥18 years who experienced STEMI within 12 h of symptom onset and agreed to participate in the trial; (2) patients in whom ≥1 of *de novo* culprit lesions in a native coronary artery were an acute infarct-related artery, requiring primary PCI; and (3) patients with a visually estimated diameter of stenosis ≥70% and reference-vessel diameter of 2.25–4.0 mm with a length of <100 mm, without excessive tortuosity or severe calcification. The exclusion criteria were as follows: (1) left ventricular ejection fraction <35%; (2) left main disease or bifurcation lesions; and (3) in-stent stenosis (≥50% luminal diameter stenosis [DS]).

### Study Endpoints

Patients were scheduled for clinical follow-up by telephone or clinic visit at 1, 6, and 12 months. Angiographic and OCT follow-up exams were performed at 6 months after the index PCI. The primary endpoint was the mean NIT of the strut coverage at 6 months after the index PCI. The safety endpoint was TLF, defined as a composite of cardiac death, target vessel-related myocardial infarction, and clinically indicated target-lesion revascularization at 12 months.

Other clinical endpoints included all-cause death; myocardial infarction; revascularization; and definite, probable, or possible ST (26). Device success was defined as the achievement of a final in-stent residual DS with the assigned study device <30% (27) and a final thrombolysis in myocardial infarction flow grade 3 by visual assessment. Procedural success was defined as device success with no periprocedural complications (28).

Quantitative coronary angiography (QCA) or OCT analyses were performed by an independent core laboratory (Zhouhe Ltd., Beijing, China). The clinical events assessment at 1 year of follow-up was performed by either clinic visit or telephone. All events were adjudicated by an independent clinical events committee.

Quantitative coronary angiography Baseline and follow-up angiograms were analyzed by two independent observers using a computer-assisted automated edge detection method (QAngio XA 7.3; Medis, Leiden, The Netherlands). QCA parameters included acute lumen gain, reference vessel diameter (RVD), minimum lumen diameter (MLD), %DS, late lumen loss (LLL), and binary restenosis (%DS ≥ 50%). All measurements were performed in-stent, in-segment, and on the 5-mm proximal and distal stent margins. We classified the thrombus into six grades (G0–G5) according to the thrombus grade (29, 30), where G0 indicated no thrombus. More details of the thrombus grade definitions are shown in Supplementary Material.

### OCT Examination and Analysis

The OCT assessments of stented coronary segments were performed using the final OCT recordings sent for offline analysis using the QIVUS software, version 3.1 (Medis, Leiden, The Netherlands). Cross-sectional OCT images were analyzed frame by frame (0.2-mm intervals). Poor-quality frames caused by residual blood or artifact, stent struts located across the ostium of side branches, and overlapping stents were excluded from OCT analysis.

The NIT on each strut was measured from the neointimal surface to the center of the strut blooming (31). A covered strut was defined if the NIT was >0 μm (32). Apposition was evaluated by measuring the distance between the center of the strut blooming and the adjacent lumen border. Malapposition was defined as a separation between strut and vessel wall with a distance greater than the thickness of the strut and polymer (Firehawk, >86 μm; Xience, >81 + 8 μm) (33).

An intraluminal defect was defined as an irregularly shaped structure, whether attached to the vessel wall or stent or not (34). The tissue coverage symmetry per frame was analyzed with the following ratio: (maximum tissue coverage thickness per frame–minimum tissue coverage thickness per frame)/maximum tissue coverage thickness per frame. This ratio had possible values of 0–1; however, the closer the ratio to 1, the greater the asymmetry of the tissue coverage (8). The neointimal healing score (32, 34, 35) was calculated at the lesion level. The healing score was based on the presence of uncovered, malapposed stent struts, and intraluminal filling defects (details are shown in the Supplementary Materials).

### Statistical Analysis

The trial was powered for testing non-inferiority for the primary endpoint (NIT) at the 6-month OCT follow-up visit after the index procedure. The expected mean NIT at 6 months post-stent implantation by Xience was 100 μm, based on previous trials (31, 33); we assumed the same mean NIT at 6 months for Firehawk. With a standard deviation of 45 μm for each group and the non-inferiority margin set at −45 μm, assuming an attrition rate of 25%, a total of 44 patients would be required to achieve an 85% power to demonstrate non-inferiority with a one-sided type α error of 0.05.

We reported categorical variables as counts and percentages; and assessed between-group differences with the chi-squared test or Fisher's exact test, as appropriate. Continuous data were expressed as mean ± standard deviation (SD) or median ± interquartile range (IQR) and were assessed by *t*-test or Mann–Whitney *U*-test. The median difference (95% confidence interval [CI]) was obtained by the Hodges–Lehman estimator based on the Mann–Whitney *U*-test. In the strut-level analysis, categorical variables and continuous variables were analyzed using multilevel logistic regression or multilevel linear regression models with random effect at the lesion level (each patient had only one target lesion), when appropriate. Multiple linear regression with backward stepwise variable selection (inclusion and elimination set at 0.05 and 0.10, respectively) was used to build the model predicting the NIT. A 2-sided *P*-value of < 0.05 was considered to be statistically significant. All analyses were conducted using SAS version 9.2 (SAS Institute Inc., Cary, NC, USA).

## Results

### Baseline Characteristics

**Figure 1:**
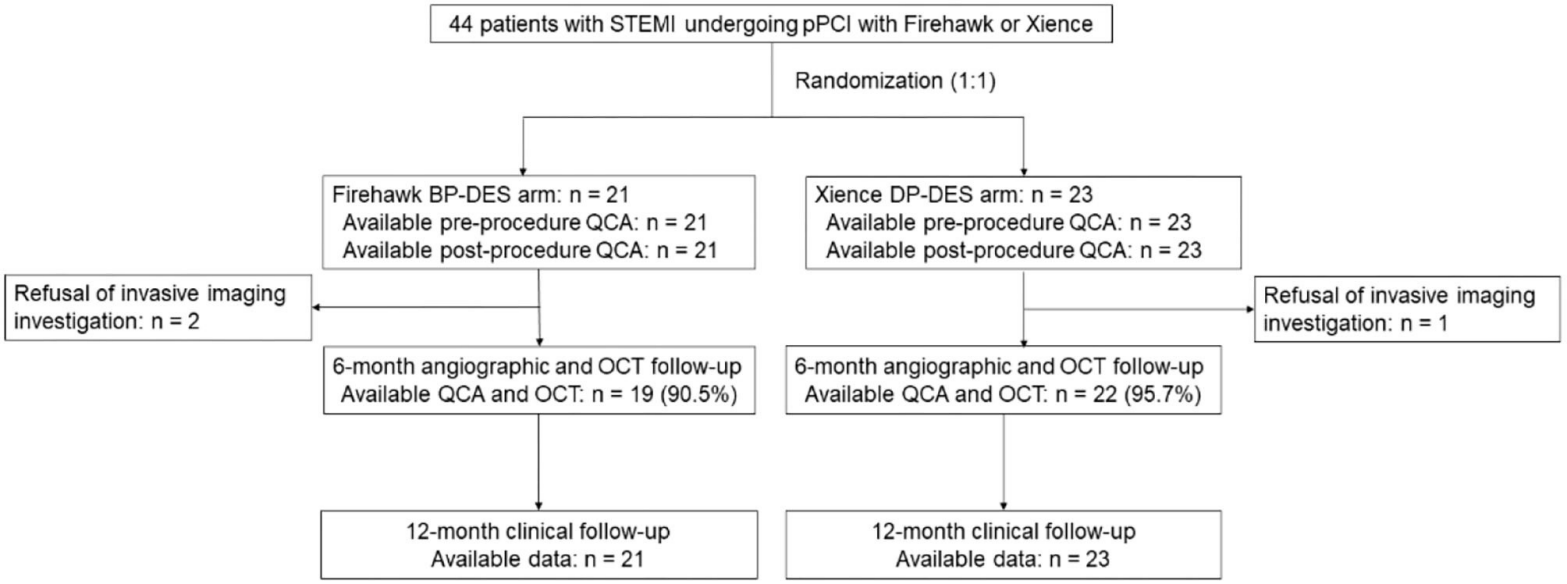
Study flowchart. BP-DES, biodegradable polymer drug-eluting stent; DP-DES, durable polymer drug-eluting stent; OCT, optical coherence tomography; pPCI, primary percutaneous coronary intervention; QCA, quantitative coronary angiography; STEMI, ST-segment elevation myocardial infarction.

Between 8 March 2019 and 9 October 2020, a total of 44 patients with STEMI were enrolled in this study (with 21 patients randomized to receive the Firehawk stent and 23 patients randomized to receive the Xience stent). The study flowchart is shown in Figure 1. At 6 months, 19 (90.5%) patients in the Firehawk group and 22 (95.7%) patients in the Xience group completed OCT follow-up. Demographic characteristics are shown in Table 1.

**Table 1:** Baseline characteristics in the Firehawk stent and Xience stent groups.

	**Firehawk (*n =* 21)**	**Xience (*n =* 23)**	**Difference (95% CI)**
Age (years)	52.14 ± 9.75	54.74 ± 11.39	−2.60 (−3.84, 9.03)
Male	19 (90.5)	21 (91.3)	−0.83 (−17.86, 16.21)
Body mass index (kg/mm^2^)	25.46 ± 2.25	25.42 ± 3.78	0.04 (−1.84, 1.92)
Diabetes mellitus	6 (28.6)	4 (17.4)	11.18 (−13.58, 35.94)
Insulin treated diabetes	0 (0.0)	2 (8.7)	−8.70 (−20.21, 2.82)
Hypertension	5 (23.8)	9 (39.1)	−15.32 (−42.33, 11.69)
Hyperlipidemia	13 (61.9)	13 (56.5)	5.38 (−23.63, 34.40)
Current smokers	14 (66.7)	14 (60.9)	5.80 (−22.56, 34.16)
Prior stroke/TIA	1 (4.8)	1 (4.4)	0.41 (−11.93, 12.76)
Family history of CVD	4 (19.1)	1 (4.4)	14.70 (−4.05, 33.45)
Previous MI	0 (0.0)	0 (0.0)	–
Previous PCI	2 (9.5)	0 (0.0)	9.52 (−3.03, 22.08)
Previous CABG	0 (0.0)	0 (0.0)	–
Peripheral artery disease	2 (9.5)	2 (8.7)	0.83 (−16.21, 17.86)
Atrial fibrillation	1 (4.7)	0 (0.0)	4.76 (−4.35, 13.87)
**Chronic kidney disease**			
*eGFR ml/ (min∙1.73m^2^)	120.08 ± 36.05	111.70 ± 29.77	8.38 (−11.89, 28.65)
History of bleeding	0 (0.0)	0 (0.0)	–
COPD	0 (0.0)	0 (0.0)	–
**Chronic heart failure**			
LVEF%	54.10 ± 5.25	54.78 ± 7.09	−0.69 (−4.47, 3.09)
**Killip class**			
Class 1	16 (76.2)	16 (69.6)	6.63 (−19.56, 32.81)
Class 2	4 (19.1)	7 (30.4)	−11.39 (−36.60, 13.83)
Class 3	0 (0.0)	0 (0.0)	–
Class 4	1 (4.8)	0 (0.0)	4.76 (−4.35, 13.87)
**Antiplatelets**			
Aspirin	21 (100.0)	22 (95.7)	4.35 (−3.99, 12.68)
Clopidogrel	3 (14.3)	4 (17.4)	−3.11 (−24.64, 18.43)
Ticagrelor	18 (85.7)	19 (82.6)	3.11 (−18.43, 24.64)

*Data are shown as n (%) or mean ± standard deviation, otherwise specified*.

^*^eGFR was estimated according to the MDRD formula.

*TIA, transient ischemic attack; CVD, cardiovascular disease; MI, myocardial infarction; PCI, percutaneous coronary intervention; CABG, coronary artery bypass grafting; COPD, chronic obstruction pulmonary disease; LVEF, left ventricular ejection fraction*.

Device and procedural characteristics are presented in Table 2. All patients had only 1 infarct-related lesion treated with the Firehawk or Xience stent. In the Firehawk arm and Xience arm, lesion complexity by the American College of Cardiology (ACC)/American Heart Association (AHA) type B2/C lesion confirmation was confirmed in 90.5 and 82.6% of patients, and thrombus was diagnosed in 85.7 and 87.0% of patients, respectively. Notably, 71.4% of patients in the Firehawk group and 69.6% of patients in the Xience group were classified as thrombus G5 cases, respectively.

**Table 2:** Device and procedural characteristics.

	**Firehawk (*n =* 21)**	**Xience (*n =* 23)**	**Difference (95% CI)**
**Number of lesions treated per patient**	1	1	–
Pain–to–balloon time, min	311 (216, 509.5)	318 (226, 506)	8.53 (−105.94, 123.01)^#^
Door–to–balloon time, min	65 (48, 85.5)	82 (57, 105)	−15.19 (−34.63, 4.24)^#^
Number of stents per patient	1.24 ± 0.54	1.30 ± 0.47	−0.07 (−0.38, 0.24)
**Target vessel**			
LAD	9 (42.9)	10 (43.5)	−0.62 (−29.92, 28.68)
LCX	4 (19.1)	2 (8.7)	10.35 (−10.01, 30.72)
RCA	8 (38.1)	11 (47.8)	−9.73 (−38.85, 19.39)
**Pre-procedural TIMI flow**			
Flow 0	17 (81.0)	17 (73.9)	7.04 (−17.54, 31.62)
Flow 1	0 (0.0)	1 (4.4)	−4.35 (−12.68, 3.99)
Flow 2	1 (8.8)	3 (13.0)	−8.28 (−24.79, 8.22)
Flow 3	3 (14.3)	3 (13.0)	1.24 (−19.09, 21.58)
**Number of vessel disease**			
Single–vessel	10 (47.6)	9 (39.1)	8.49 (−20.74, 37.71)
Multi–vessel disease	11 (52.4)	14 (60.9)	−8.49 (−37.71, 20.74)
Two–vessel	6 (28.6)	9 (39.1)	−10.56 (−38.33, 17.21)
Three–vessel	5 (23.8)	5 (21.7)	2.07 (−22.75, 26.89)
**AHA/ACC lesion classification**			
A/B1	2 (9.5)	4 (17.4)	−7.87 (−27.81, 12.07)
B2/C	19 (90.5)	19 (82.6)	–
Thrombus present	18 (85.7)	20 (87.0)	−1.24 (−21.58, 19.09)
Thrombus aspirated	12 (57.1)	11 (47.8)	9.32 (−20.09, 38.72)
**Classification of thrombus**			
Grade 0	1 (4.8)	2 (8.70)	−3.93 (−18.62, 10.75)
Grade 1	0 (0.0)	1 (4.35)	−4.35 (−12.68, 3.99)
Grade 2	1 (4.8)	0 (0.0)	4.79 (−4.35, 13.87)
Grade 3	1 (4.8)	0 (0.0)	4.79 (−4.35, 13.87)
Grade 4	3 (14.3)	4 (17.4)	−3.11 (−24.64, 18.43)
Grade 5	15 (71.4)	16 (69.6)	2.23 (−34.54, 30.08)
**SYNTAX score**			
Baseline score	14.14 ± 7.01	14.02 ± 7.98	0.12 (−4.44, 4.68)
Residual SYNTAX score	2.33 ± 3.37	1.65 ± 2.96	0.68 (−1.26, 2.62)
**Complete revascularization (CR)**	11 (52.4)	13 (56.5)	−4.14 (−33.58, 25.30)
One–staged CR	5 (45.5)	6 (46.2)	−8.07 (−36.83, 20.68)
Elective CR	6 (54.5)	7 (53.8)	8.07 (−20.68, 36.83)
Non–Complete revascularization	10 (47.6)	10 (43.5)	4.14 (−25.30, 33.58)
Post–procedural TIMI flow = 3	21 (100.0)	23 (100.0)	–
**Pre–balloon dilation performed**			
Balloon nominal diameter (mm)	2.14 ± 0.55	2.43 ± 0.27	0.29 (0.02, 0.56)
Maximum pressure (atm)	12.48 ± 3.22	12.70 ± 1.55	0.22 (−1.36, 1.80)
Pre–balloon usage	20 (95.2)	23 (100.0)	−4.76 (−13.87, 4.35)
**Stent implantation**			
Stent nominal diameter (mm)	3.16 ± 0.39	3.25 ± 0.35	−0.09 (−0.29, 0.11)
Stent length (mm)	30.23 ± 7.07	32.67 ± 5.86	−2.44 (−5.90, 1.03)
Maximum dilation pressure (atm)	10.74 ± 1.98	9.95 ± 2.40	−0.79 (−2.14, 0.56)
**Post–dilation performed**			
Balloon nominal diameter (mm)	3.26 ± 0.30	3.40 ± 0.45	0.14 (−0.09, 0.37)
Maximum dilation pressure (atm)	19.24 ± 2.32	18.96 ± 2.16	−0.28 (−1.65, 1.09)
Non–compliant balloon usage	21 (100.0)	23 (100.0)	–
Device success	21 (100.0)	23 (100.0)	–
Technical success	21 (100.0)	23 (100.0)	–

*Data are shown as n (%) or mean ± SD or median ± interquartile range (IQR), otherwise specified*.

*^#^The median difference (95% CI) was obtained by the Hodges–Lehman estimator based on Mann–Whitney U-test*.

*LAD, Left anterior descending coronary artery; LCX, Left circumflex coronary artery; RCA, right coronary artery; TIMI, thrombolysis in myocardial infarction; ACC/AHA, American College of Cardiology/American Heart Association*.

### Results of QCA Measurements

**Figure 2:**
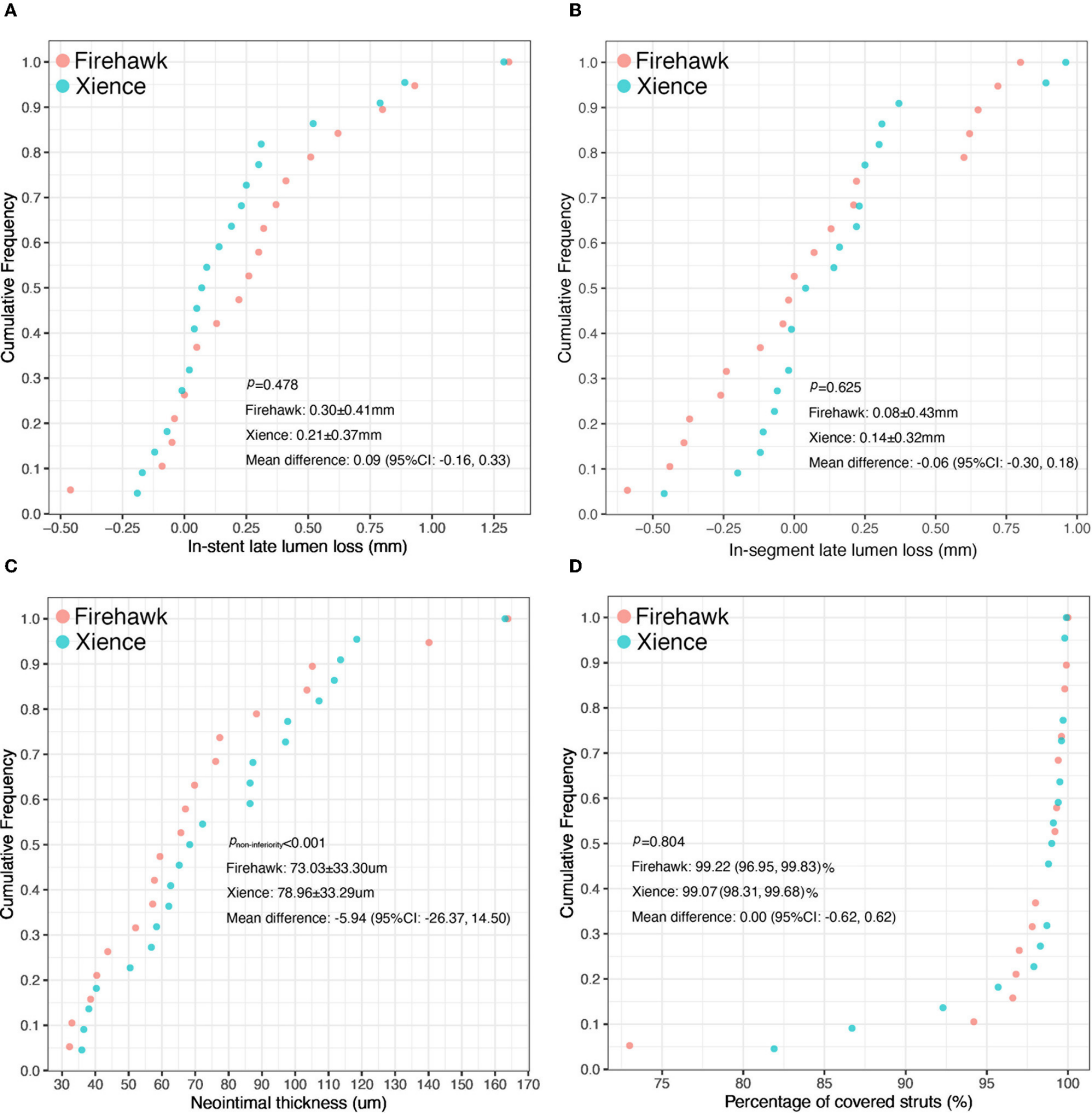
Cumulative frequency distribution curves of in-stent late lumen loss **(A)**, in-segment late lumen loss **(B)**, the mean neointimal thickness **(C)**, and the percentage of covered stent struts **(D)** at the 6-month OCT and angiographic follow-up.

The results of the QCA analysis are reported in Table 3. No significant difference was observed between the two groups in terms of preprocedural RVD, MLD, and %DS. The Firehawk group had smaller diameters of the post-procedural RVD and MLD compared with the Xience group (in-stent analyses). At the 6-month angiographic follow-up visit, the RVD was still smaller in the Firehawk group compared with the Xience group (2.97 ± 0.37 mm vs. 3.30 ± 0.43 mm, *P* = 0.012); however, the LLL and binary restenosis showed no significant differences between the two groups (Table 3, Figure 2A, in-stent analyses). The results of the in-segment analyses were similar to those of the in-stent analyses (Table 3, Figure 2B).

**Table 3:** Quantitative coronary angiographic analysis.

	**Proximal edge**			**In–stent**			**Distal edge**			**In–segment**		
	**Firehawk**	**Xience**	***P*-value**	**Firehawk**	**Xience**	***P*-value**	**Firehawk**	**Xience**	***P-*value**	**Firehawk**	**Xience**	***P*-value**
**Reference vessel diameter, mm**												
Pre–procedure	NA	NA	NA	2.95 ± 0.36	3.13 ± 0.42	0.133	NA	NA	NA	2.90 (2.68, 3.16)	3.05 (2.76, 3.37)	0.124
Post–procedure	3.31 ± 0.30	3.51 ± 0.37	0.054	3.07 (2.82, 3.29)	3.41 (3.24, 3.55)	0.004	2.69 ± 0.38	2.86 ± 0.45	0.179	2.86 ± 0.39	3.18 ± 0.50	0.023
At 6 months	3.34 ± 0.32	3.45 ± 0.42	0.339	2.97 ± 0.37	3.30 ± 0.43	0.012	2.72 ± 0.42	2.92 ± 0.47	0.160	2.99 ± 0.39	3.19 ± 0.51	0.157
**Mean lumen diameter, mm**												
Post-procedure	3.33 ± 0.40	3.46 ± 0.49	0.339	2.98 ± 0.28	3.14 ± 0.33	0.087	2.69 ± 0.36	2.87 ± 0.47	0.176	2.95 ± 0.24	3.13 ± 0.34	0.046
At 6 months	3.33 ± 0.46	3.24 ± 0.53	0.604	2.94 ± 0.36	3.11 ± 0.36	0.137	2.62 ± 0.37	2.86 ± 0.48	0.090	2.93 ± 0.30	3.11 ± 0.36	0.090
**Minimal lumen diameter, mm**												
Pre-procedure	NA	NA	NA	0.00 (0.00, 0.27)	0.00 (0.00, 0.01)	0.318	NA	NA	NA	0.00 (0.00, 0.27)	0.00 (0.00, 0.01)	0.652
Post-procedure	3.17 ± 0.42	3.38 ± 0.55	0.160	2.65 ± 0.29	2.89 ± 0.31	0.015	2.57 ± 0.39	2.77 ± 0.49	0.153	2.31 ± 0.33	2.61 ± 0.54	0.039
Acute gain	NA	NA	NA	2.46 ± 0.47	2.72 ± 0.45	0.064	NA	NA	NA	2.12 ± 0.46	2.44 ± 0.62	0.061
At 6 months	3.13 ± 0.57	3.14 ± 0.57	0.975	2.38 ± 0.50	2.67 ± 0.45	0.057	2.52 ± 0.40	2.73 ± 0.53	0.171	2.24 ± 0.47	2.44 ± 0.53	0.212
Late lumen loss (LLL)	0.02 ± 0.39	0.21 ± 0.30	0.077	0.26 (0.00, 0.51) 0.30 ± 0.41	0.08 (−0.01, 0.30) 0.21 ± 0.37	0.284 0.478	0.07 ± 0.27	0.05 ± 0.28	0.765	0.08 ± 0.43	0.14 ± 0.32	0.625
**Diameter stenosis, %**												
Pre–procedure	NA	NA	NA	100.00 (80.66, 100.00)	100.00 (99.48, 100.00)	0.378	NA	NA	NA	100.00 (91.38, 100.00)	100.00 (99.48, 100.00)	0.652
Post–procedure	3.08 (−0.01, 7.50)	2.69 (−2.61, 7.90)	0.698	12.54 ± 4.72	12.87 ± 4.52	0.812	3.70 ± 12.08	3.12 ± 8.84	0.856	15.98 (14.48, 23.96)	16.32 (12.01, 22.21)	0.474
At 6 months	6.31 ± 13.25	9.26 ± 10.21	0.426	20.38 ± 11.12	19.29 ± 9.49	0.737	6.81 ± 9.22	6.37 ± 11.59	0.895	25.24 ± 10.37	23.77 ± 10.20	0.651
**Binary restenosis**												
At 6 months	1/19 (5.26)	0 (0.00)	0.463	0 (0.00)	0 (0.00)		0 (0.00)	0 (0.00)		1/19 (5.26)	0 (0.00)	0.463

*Data are shown as n (%) or mean ± SD or median ± interquartile range (IQR), otherwise specified*.

### Results of OCT Measurements

**Figure 3:**
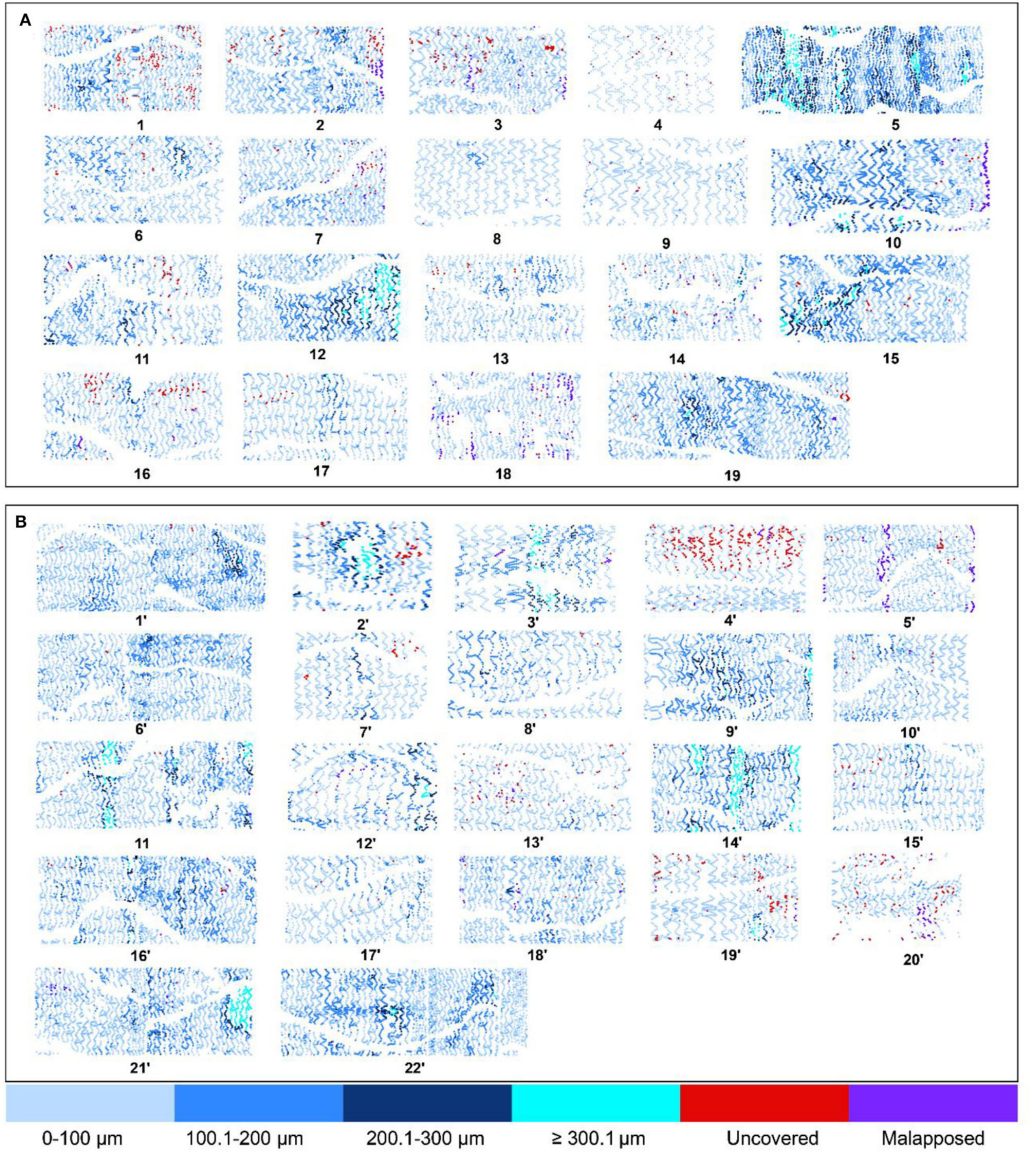
The neointimal thickness and coverage or malapposition of stent status in individual stents at 6 months. The spread-out sheets demonstrate the coverage stent status of individual stents at 6 months. **(A)** Demonstrates the coverage and malapposition status of the Firehawk stents, while **(B)** demonstrates the coverage and malapposition status of the Xience stents. Struts are color-coded according to their coverage status. Uncovered struts are depicted as red; malapposed struts are depicted as purple; and covered struts are depicted as blue and cyan, with deepening color of blue indicative of a thicker neointima (light blue color indicates a neointimal thickness ≤ 100 μm, sustained blue indicates a neointimal thickness 100.1–200 μm, navy blue indicates a neointimal thickness 200.1–300 μm, and cyan indicates a neointimal thickness ≥300.1 μm). The x-axis indicates the position of the strut in the individual cross-section ranging from 0° to 360°, whereas the y-axis represents the cross-section of the stent from distal (left) to proximal (right).

The OCT results are shown in Table 4, Figure 3. At the strut level, the mean NIT by OCT was not significantly different between the groups (Firehawk, 73.03 ± 33.30 μm; Xience, 78.96 ± 33.29 μm), meeting the primary endpoint of non-inferiority (absolute difference: −5.94 [one-sided 95% lower confidence bound: −23.09]; *P*_non−inferiority_ < 0.001) (Figure 2C). The percentage of covered struts was high and not significantly different between the 2 groups (Firehawk, 99.22 [96.95, 99.83]%; Xience, 99.07 [98.31, 99.68]%; difference: 0.00 [95%CI: −0.62, 0.62]; *P* = 0.804) (Figure 2D). The percentage of uncovered struts was 0.55 [0.08, 1.32]% in the Firehawk arm and 0.40 [0.21, 1.19]% in the Xience arm (difference: −0.03 [95%CI: −0.39, 0.34]; *P* = 0.804). The percentage of malapposed struts was low (<0.2%) in both groups.

**Table 4:** Six–month qualitative and quantitative optical coherence tomography analysis (as treated analysis).

	**Firehawk (*n =* 19)**	**Xience (*n =* 22)**	**Difference (95% CI)**	***P*-value**
**Strut level analysis**				
Mean neointimal thickness, μm	73.03 ± 33.30	78.96 ± 33.29	−5.94 (−26.37, 14.50)	<0.001*
Total analysis struts	37,822	45,033		
Number of struts per cross–section	11.09 ± 2.19	10.26 ± 1.77	0.84 (−0.44, 2.11)	0.191
Number of covered struts	1,799 (1,324, 2,359)	1,532 (1,231, 2,595)	50.5 (−469, 570)	0.666
Percentage of covered struts	99.22 (96.95, 99.83)	99.07 (98.31, 99.68)	0.00 (−0.62, 0.62)	0.804
Number of uncovered struts	10 (3,18)	10.5 (4,18)	−1.5 (−8, 5)	0.647
Percentage of uncovered struts	0.55 (0.08, 1.32)	0.40 (0.21, 1.19)	−0.03 (−0.39, 0.34)	0.804
Number of malapposed struts	3 (0, 35)	3 (0, 18)	0.5 (−2, 3)	0.789
Percentage of malapposed struts	0.17 (0.00, 1.52)	0.17 (0.00, 0.69)	0.06 (−0.14, 0.25)	0.662
Number of Uncovered malapposed struts	0 (0, 0)	0 (0, 0)	0 (0, 0)	0.452
Percentage of Uncovered malapposed struts	0.00 (0.00, 0.00)	0.00 (0.00, 0.00)	0.00 (0.00, 0.00)	0.452
**Cross–section level analysis**				
Number of cross–sections per lesion	174 (148, 191)	184 (139, 288)	10 (−55, 35)	0.583
Percentage analysis frames	100.00 (100.00, 100.00)	100.00 (98.88, 100.00)	0.00 (0.00, 0.00)	0.187
Mean lumen area	7.39 ± 1.73	8.00 ± 1.97	−0.61 (−1.77, 0.56)	0.299
Minimal lumen area	5.41 ± 1.67	5.93 ± 2.11	−0.52 (−1.72, 0.67)	0.382
Mean stent area	7.72 ± 1.65	8.46 ± 2.03	−0.74 (−1.90, 0.42)	0.206
Minimal stent area	5.96 ± 1.61	6.84 ± 2.01	−0.88 (−2.02, 0.27)	0.130
Neointimal hyperplasia area	0.33 ± 0.48	0.46 ± 0.30	−0.13 (−0.39, 0.13)	0.307
**Lesion–level analysis**				
Healing score	1.56 (0.23, 5.74)	2.12 (0.91, 3.81)	−0.17 (−1.54, 1.20)	0.647
Mean Lumen volume	243.51 (190.05, 326.49)	257.72 (196.53, 394.67)	−36.26 (−115.03, 42.52)	0.505
Mean Stent volume	257.73 (215.23, 332.87)	279.45 (200.79, 407.55)	−41.01 (−127.75, 45.73)	0.410
Neointimal volume	11.35 (2.75, 25.18)	16.96 (7.01, 30.28)	−5.23 (−15.25, 4.78)	0.353
Mean Lumen diameter	3.04 ± 0.37	3.16 ± 0.40	−0.12 (−0.37, 0.12)	0.314
Minimal Lumen diameter	2.59 ± 0.43	2.70 ± 0.52	−0.11 (−0.41, 0.19)	0.463
Mean stent diameter	3.11 ± 0.34	3.25 ± 0.41	−0.14 (−0.38, 0.09)	0.229
Minimal stent diameter	2.73 ± 0.38	2.92 ± 0.44	−0.19 (−0.45, 0.07)	0.150
Tissue coverage symmetry ratio	0.72 (0.63, 0.75)	0.72 (0.68, 0.74)	−0.01 (−0.05, 0.03)	0.990

^*^Tested for non–inferiority.

At the cross-section level, the neointimal hyperplasia areas were 0.33 ± 0.48 mm^2^ and 0.46 ± 0.30 mm^2^ in the Firehawk and Xience arms, respectively (difference: −0.13 [95%CI: −0.39, 0.13]; *P* = 0.307). At the lesion level, the healing score at 6 months did not differ between the Firehawk arm and the Xience arm (Firehawk, 1.56 [0.23, 5.74]; Xience, 2.12 [0.91, 3.81], difference: −0.17 [95%CI: −1.54, 1.20]; *P* = 0.647). The tissue symmetry ratio was comparable between the two groups (Firehawk, 0.72 [0.63, 0.75]; Xience, 0.72 [0.68, 0.74], difference: −0.01 [95%CI: −0.05, 0.03]; *P* = 0.990).

### Clinical Outcomes

The clinical events assessment at 1-year follow-up was performed either during a clinic visit (68.2%) or by telephone (31.8%). At 1 year, no definite, probable, or possible ST occurred in any of the 44 patients. One patient in the Firehawk group underwent clinically indicated target lesion revascularization at 6 months. No other major adverse events occurred in either of the groups.

### Risk Factors of the NIT

The independent risk factors of NIT (Table 5) included the demographic characteristics of body mass index (β = −0.07, *P* = 0.001) and hyperlipidemia (β = −0.23, *P* = 0.047) and the procedural characteristics of ACC/AHA-type B2/C lesions (β = −1.08, *P* = 0.009), thrombus G3 (β = 1.45, *P* = 0.009), G4 (β = 1.27, *P* = 0.004), and G5 (β = 1.23, *P* = 0.014), thrombus aspiration (β = −0.28, *P* = 0.019), and postdilation pressure (β = 0.10, *P* = 0.001) (R^2^ = 0.690, *P* < 0.001).

**Table 5:** Independent risk factors of neointimal thickness (*n* = 41).

**Baseline risk factors**	**Multiple model**		
	**β**	**Std. β**	***P-*value**
**Demographic characteristics**			
Body mass index (kg/mm^2^)	−0.07	−0.45	0.001
Hyperlipidemia	−0.23	−0.26	0.047
**Procedural characteristics**			
B2/C lesion	−1.08	−0.83	0.009
Thrombus G1*	−0.04	−0.02	0.916
Thrombus G2*	−0.08	−0.03	0.849
Thrombus G3*	1.45	0.52	0.009
Thrombus G4*	1.27	1.05	0.004
Thrombus G5*	1.23	1.27	0.014
Thrombus aspiration	−0.28	−0.33	0.019
Post-dilation pressure	0.10	0.50	0.001

*Thrombus G0 as the reference.

*The considered variables included the demographic variables (Age, Sex, BMI), procedural variables (target vessel, pre-procedural TIMI flow, two-vessel disease, three vessel disease, A/B1, B2/C, thrombus aspiration, classification of thrombus, pre-balloon dilation pressure and diameter, post-balloon dilation pressure and diameter, stent length, diameter, and pressure), complications (diabetes mellitus, hypertension, hyperlipidemia), medical history (current smokers, prior stroke/TIA, family history of CVD, previous MI, previous PCI, previous CABG), and SYNTAX score, eGFR, LVEF, Killip class*.

## Discussion

Our findings can be summarized as follows: (1) the Firehawk stent was non-inferior to the Xience stent in terms of the mean NIT at 6 months after the index procedure in patients presenting with STEMI, and the percentage of uncovered struts was 0.55% in the Firehawk arm and 0.40% in the Xience arm; (2) at 1 year of follow-up, one patient in the Firehawk group experienced a clinically indicated target lesion revascularization, but no other TLF or definite, probable, or possible ST events occurred in either group; and (3) body mass index, hyperlipidemia, B2/C lesions, thrombus G3–G5, thrombus aspiration, and postdilation pressure are independent risk factors of NIT.

The performance of Xience stents has been well-documented. In the angiographic and OCT TROFI II substudy, the mean NIT after Xience implantation in patients with STEMI at 6 months was 100 μm with postdilation, and the percentage of covered struts was 97.6% (36). In the ISAR-ABSORB MI trial, the NIT at 6–8 months was 76.7 μm, and the percentage of struts coverage was 91.5% (37). Sawada et al. found that the NIT at 7 months was 94.8 μm, and the percentage of strut coverage was 97.1% (33). In our study, the performance of the Xience group in terms of NIT and stent strut coverage was similar to the results of these previous studies.

Compared with the Xience stent, Firehawk has a unique design, in which the biodegradable polymer is embedded in grooves on the abluminal strut surface, which can reduce polymer exposure to the vessel wall and allow timed and targeted sirolimus release, thereby reducing drug concentrations in the vessel wall (38, 39). The drug dose of Firehawk (0.3 μg/mm^2^) is lower than that of Xience (1.0 μg/mm^2^) and other stents (Supplementary Table). Firehawk has been assessed in a large-scale randomized controlled trial (23) and an OCT study (24). In the OCT substudy of the TARGET All Comers trial (24), a total of 36 cases of stable angina, unstable angina, and patients with non-STEMI were enrolled. At 3 months after stent implantation, the NITs were 75.5 and 82.3 μm, and the percentages of covered struts were 99.9 and 100% in the Firehawk and Xience arms, respectively. The healing response of the Firehawk stent in patients with STEMI remains unclear. It has been reported that patients with STEMI experience greater inflammation and delayed endothelialization compared with the patients with stable CAD after DES implantation (5, 12, 13). Previous literature has suggested that more than 5.9% of incomplete endothelialization struts were associated with an increased ST rate (40); therefore, we implemented a 6-month OCT follow-up to evaluate the vascular healing, which might be the time when >95% endothelialization rate might be expected in patients with STEMI. We found that, at 6 months, the NITs of the Firehawk and Xience groups were 73.0 and 79.0 μm, respectively, and the strut coverage values were 99.2 and 99.1%, respectively.

It has been documented that the overwhelming growth of neointima can lead to in-stent restenosis (41). Therefore, it is crucial to identify the contributing factors of NIT not only to improve vascular healing but also to avoid excessive neointimal hyperplasia. In this study, we found that the independent risk factors for the NIT were body mass index, hyperlipidemia, B2/C lesions, thrombus G3–G5, thrombus aspiration, and postdilation pressure. A previous report has suggested that the relative position of the overlying stent struts within any thrombus could modulate arterial drug distribution for DES (42). It was assumed that when the thrombus was interposed between the strut and artery, a barrier to transport was created, which could decrease arterial drug uptake. The arterial drug distribution might have a negative correlation with the distance and size of a thrombus between the stent strut and the artery (42). In our analyses, patients with greater thrombus burdens during the index PCI for STEMI had thicker neointimal hyperplasia at 6 months, while patients with thrombus aspiration had thinner neointimal hyperplasia. These results supported the idea that achieving a lower thrombus burden during primary PCI for STEMI would result in a thinner neointimal hyperplasia.

We found that lesions with higher postdilation pressures had thicker neointimal hyperplasia. This finding was in line with previous literature (36), showing that the NIT tends to be greater in lesions with postdilation compared with those without. This result might be explained by the fact that overstretching of the coronary artery could potentially provoke inflammation and, therefore, lead to neointimal hyperplasia (43). This result suggests that appropriate postdilation pressures rather than higher pressures should be used to ensure stent apposition.

In the STEMI population, the DAPT-STEMI trial showed that DAPT for 6 months was non-inferior to DAPT for 12 months (44). The SMART-DATE trial did not concur with this result, and the investigators found an increased risk of myocardial infarction with 6 months of DAPT (45). Suwannasom et al. evaluated vascular healing of polymer-free DESs by serial follow-up at 3 and 6 months with OCT (46). In their study, whether DAPT was discontinued at 4 or 7 months depended on the vascular healing. Throughout the 8-month follow-up period, no definite, probable, or possible ST was reported, and the authors concluded that DAPT cessation could be safe after the confirmation of favorable vascular healing evaluated by OCT. Both the ACC/AHA and European Society of Cardiology guidelines recommend a 6-month DAPT duration in the STEMI population with a high bleeding risk (47, 48). In our study, in both groups, approximately 99% of stent struts, but not 100%, were covered at 6 months after the primary PCI. The safety and efficacy of shortening DAPT to 6 months for Firehawk or Xience stent recipients remain to be scrutinized in large-scale studies, and whether we can shorten the DAPT duration according to the vascular healing evaluated by OCT still needs further verification. Nevertheless, a study investigating the safety of short-term DAPT in Firehawk stent recipients with acute coronary syndrome, namely, the TARGET FIRST Clinical Trial, is ongoing in Europe (NCT04753749).

### Limitations

First, the number of enrolled patients was relatively small and recruited for non-inferiority testing. Thus, any comparison beyond the primary analysis has limited statistical power and requires cautious interpretation. Furthermore, the results from the multivariable analysis related to the risk factors favoring NIT are only speculative, as the number of enrolled patients was relatively small. Second, 90% of subjects were men in this study. Third, we excluded the cases of bifurcation, severe calcification, or tortuous lesions; thus, the nature of subjects enrolled in the current study might be skewed toward a low-risk population, and our findings should not be generalized to patients presenting with these lesions. Fourth, baseline OCT examinations at index procedures were not performed; therefore, it would be difficult to identify whether stent strut malapposition presenting at follow-up was due to the index procedure or late acquisition. Fifth, the non-serial design did not allow the assessment of serial changes of coverage over time. Finally, the predefined non-inferiority margin might be considered, in retrospect, to be too wide. However, with a *post-hoc* non-inferiority margin of −25 μm, the non-inferiority testing criterion would still be met (*P*_non−inferiority_ = 0.002).

## Conclusion

This study showed that the Firehawk was non-inferior to Xience at 6 months in terms of NIT as accessed by OCT in the STEMI population. No significant differences were observed between patients with STEMI treated with Firehawk and Xience stents in terms of incomplete stent strut neointimal coverage and stent strut malapposition. Large-scale trials with multiple series OCT examinations and longer follow-up periods are warranted in the future.

## Data Availability Statement

The raw data supporting the conclusions of this article will be made available by the authors, without undue reservation.

## Ethics Statement

The studies involving human participants were reviewed and approved by Xijing Hospital Ethics Committees. The patients/participants provided their written informed consent to participate in this study.

## Author Contributions

LT, CG, and YH conceived and designed the study. JLiu, CL, JK, XL, YL, JLi, and SC contributed to the data collection. JLiu, WY, FM, and JW performed the data extraction and analysis. MZ was a coordinator. JZ and ZZ contributed to the data analysis and interpretation and the critical revision of the manuscript. YH and RW drafted the manuscript. All authors contributed to the article and approved the submitted version.

## Funding

This trial was an investigator-initiated study sponsored by the Program for National Science Funds of China (Grant No. 82100362), the National Key R&D Program of China (Grant No. 2018YFA0107400), and an unrestricted grant from MicroPort Medical Co., Ltd. (Shanghai, China). MicropPort had no role in the study design, data collection, data analyses, or interpretation of the study data nor was the company involved in the decision to publish the final manuscript. The principal investigators and authors had complete scientific freedom.

## Conflict of Interest

MZ is an employee of MicroPort Medical (Group), Shanghai, China. The remaining authors declare that the research was conducted in the absence of any commercial or financial relationships that could be construed as a potential conflict of interest.

## Publisher's Note

All claims expressed in this article are solely those of the authors and do not necessarily represent those of their affiliated organizations, or those of the publisher, the editors and the reviewers. Any product that may be evaluated in this article, or claim that may be made by its manufacturer, is not guaranteed or endorsed by the publisher.
