# Supplementary material for: A Randomized Comparison of the Healing Response Between the Firehawk Stent and the Xience Stent in Patients With ST-Segment Elevation Myocardial Infarction at 6 Months of Follow-Up (TARGET STEMI OCT China Trial): An Optical Coherence Tomography Study
Source: Front Cardiovasc Med. 2022 Jun 1;9:895167. doi: 10.3389/fcvm.2022.895167 (PMC9198262; doi:10.3389/fcvm.2022.895167)
Supplement: Supplementary file 1 [file Data_Sheet_1.docx]

**Study eligibility criteria**

**Inclusion criteria:**

1. The patient is at least 18 years of age;
2. Evidence of ST-segment elevation myocardial infarction within 12 hours of symptom onset:
3. 1mm of ST-segment elevation in 2 contiguous leads or
4. new left bundle branch blocks or
5. true posterior myocardial infarction
6. At least one of *de novo* culprit lesions in a native coronary artery must be an acute-infarct-related artery and requiring primary PCI;
7. Lesion(s) must have a visually estimated diameter stenosis no less than 70%;
8. Reference-vessel diameter (RVD) must be visually estimated ≥2.5mm and ≤4.0mm and the vessel length must be no more than 100mm;
9. Without excessive tortuosity or severe calcification;
10. Written informed consent;
11. Have coronary artery bypass graft surgery contraindications;
12. The patient and the patient’s physician agree to the follow-up visits including angiographic and OCT follow-up and OCT at 6 months.

**Exclusion criteria:**

1. Cardiogenic shock;
2. Left ventricular ejection fraction (LVEF) <35%;
3. Known hypersensitivity or contraindication to aspirin, P2Y_12_ inhibitor, sirolimus, everolimus, contrast medium, heparin or cobalt metal;
4. Per investigator judgement that may cause non-compliance with the protocol or confound the data interpretation or is associated with a limited life expectancy (less than 1 year);
5. Pregnant or breastfeeding woman or woman in fertile period not taking adequate contraceptives;
6. Renal insufficiency with elevated serum creatinine >2.0mg/dl or undergoing dialysis therapy;
7. Active bleeding at the time of inclusion;
8. Reference vessel diameter <2.25mm or >4.0mm, vessel length >100mm;
9. True bifurcation lesion (both main and branch vessel need stents implantation);
10. Left main disease;
11. In-stent stenosis (≥50% luminal diameter stenosis);
12. Unsuitable for 6 months OCT follow-up;
13. Participation in another clinical trial (12 months after index procedure);
14. Those who are not suitable to attend this trial after the evaluation by the doctor.

**Classification of the thrombus**

1. In thrombus grade 0 (G0), no cineangiographic characteristics of thrombus are present;
2. In thrombus grade 1 (G1), possible thrombus is present, with such angiography characteristics as reduced contrast density, haziness, irregular lesion contour, or a smooth convex meniscus at the site of total occlusion suggestive but not diagnostic of thrombus;
3. In thrombus grade 2 (G2), there is definite thrombus, with greatest dimensions ≤1/2 the vessel diameter;
4. In thrombus grade 3 (G3), there is definite thrombus but with greatest linear dimension >1/2 but <2 vessel diameters;
5. In thrombus grade 4 (G4), there is definite thrombus, with the largest dimension ≥2 vessel diameters;
6. In thrombus grade 5 (G5), there is total occlusion.

**The healing score formula**

The %ILD is calculated as a percentage of intraluminal defect volume divided by the stent volume. The percentage of malapposed and uncovered struts (%MU) was (the number of malapposed and uncovered strut) / (the total number of struts)×100%. The percentage of uncovered strut (%U) was calculated as (the number of uncovered struts) / (the total number of struts)×100%, the percentage of malapposed struts (%M) was (the number of malapposed struts) / (the total number of struts)×100%.

1. Presence of intraluminal filling defect (% intraluminal defect, %ILD) is assigned a weight of “4”
2. Presence of malapposed and uncovered struts (% malapposed/uncovered, %MU) is assigned a weight of “3”
3. Presence of uncovered struts alone (% uncovered, %U) is assigned a weight of “2”
4. Presence of malapposed struts alone (% malapposed, %M) is assigned a weight of “1”

Neointimal healing score = (%ILD×4) + (%MU×3) + (%U×2) + (%M×1).

**Fig. Illustration of the neointimal healing score**


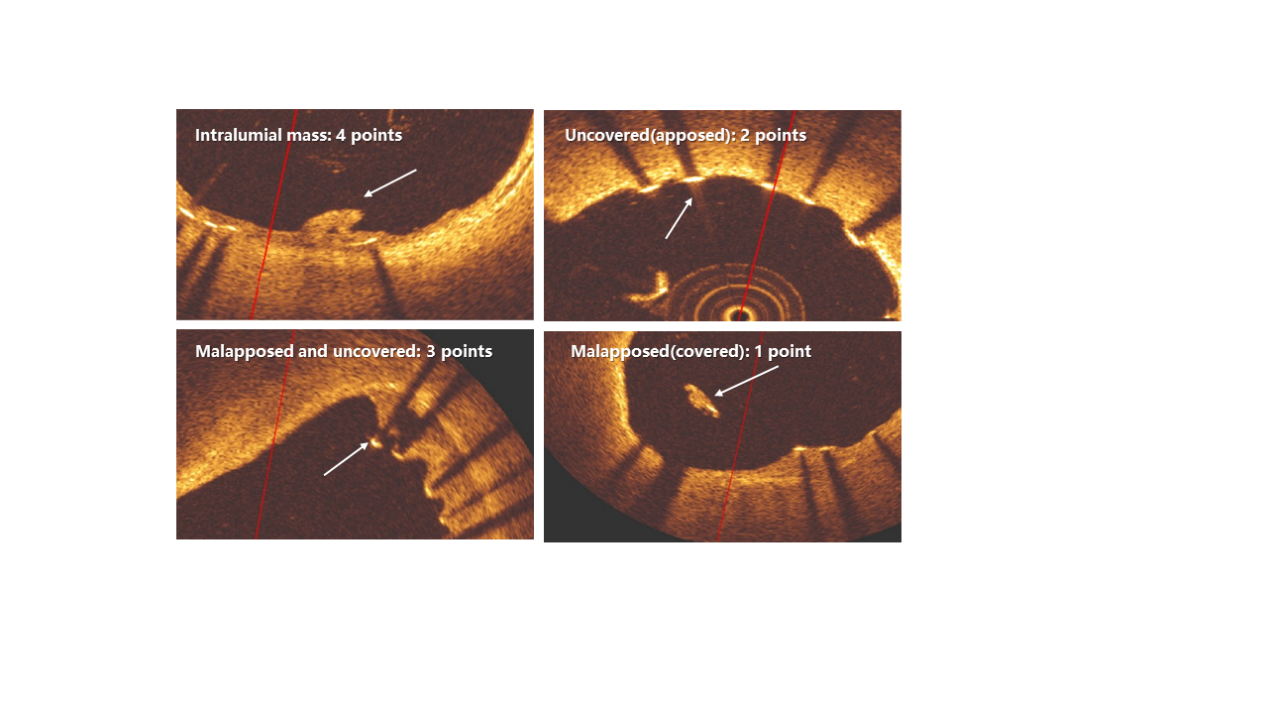


Arrows point to the intraluminal mass and to the uncovered and malapposed stent struts.

| **Supplementary Table**  The drug concentration of new-generation stents | | |
| --- | --- | --- |
| Stent | Drug | Drug Concentration |
| Firehawk | Sirolimus | 0.3 μg/mm^2^ |
| Xience family | Everolimus | 1.0 μg/mm^2^ |
| Resolute Integrity Onyx | Zotarolimus | 1.6 μg/ mm^2^ |
| Promus Element Premier | Everolimus | 1.0 μg/ mm^2^ |
| Synergy | Everolimus | 1.0 μg/mm^2^ |
| Orsiro | Sirolimus | 1.4 μg/mm^2^ |
| BioMime | Sirolimus | 1.25 μg/mm^2^ |
| Stentys | Sirolimus | 1.4 μg/ mm^2^ |

**Supplementary CONSORT checklist**


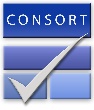
CONSORT 2010 checklist of information to include when reporting a randomised trial*

| Section/Topic | Item No | Checklist item | Reported on page No |
| --- | --- | --- | --- |
| Title and abstract | | | |
|  | 1a | Identification as a randomised trial in the title | 1 |
|  | 1b | Structured summary of trial design, methods, results, and conclusions (for specific guidance see CONSORT for abstracts) | 2 |
| Introduction | | | |
| Background and objectives | 2a | Scientific background and explanation of rationale | 3 |
|  | 2b | Specific objectives or hypotheses | 3 |
| Methods | | | |
| Trial design | 3a | Description of trial design (such as parallel, factorial) including allocation ratio | 4 |
|  | 3b | Important changes to methods after trial commencement (such as eligibility criteria), with reasons | NA |
| Participants | 4a | Eligibility criteria for participants | 4 |
|  | 4b | Settings and locations where the data were collected | 4 |
| Interventions | 5 | The interventions for each group with sufficient details to allow replication, including how and when they were actually administered | 4-5 |
| Outcomes | 6a | Completely defined pre-specified primary and secondary outcome measures, including how and when they were assessed | 4-5 |
|  | 6b | Any changes to trial outcomes after the trial commenced, with reasons | NA |
| Sample size | 7a | How sample size was determined | 5-6 |
|  | 7b | When applicable, explanation of any interim analyses and stopping guidelines | NA |
| Randomisation: |  |  |  |
| Sequence generation | 8a | Method used to generate the random allocation sequence | 4 |
|  | 8b | Type of randomisation; details of any restriction (such as blocking and block size) | 4 |
| Allocation concealment mechanism | 9 | Mechanism used to implement the random allocation sequence (such as sequentially numbered containers), describing any steps taken to conceal the sequence until interventions were assigned | 4 |
| Implementation | 10 | Who generated the random allocation sequence, who enrolled participants, and who assigned participants to interventions | NA |
| Blinding | 11a | If done, who was blinded after assignment to interventions (for example, participants, care providers, those assessing outcomes) and how | NA |
|  | 11b | If relevant, description of the similarity of interventions | NA |
| Statistical methods | 12a | Statistical methods used to compare groups for primary and secondary outcomes | 5-6 |
|  | 12b | Methods for additional analyses, such as subgroup analyses and adjusted analyses | NA |
| Results | | | |
| Participant flow (a diagram is strongly recommended) | 13a | For each group, the numbers of participants who were randomly assigned, received intended treatment, and were analysed for the primary outcome | 7 |
|  | 13b | For each group, losses and exclusions after randomisation, together with reasons | 7 |
| Recruitment | 14a | Dates defining the periods of recruitment and follow-up | 7 |
|  | 14b | Why the trial ended or was stopped | 7 |
| Baseline data | 15 | A table showing baseline demographic and clinical characteristics for each group | Table 1 |
| Numbers analysed | 16 | For each group, number of participants (denominator) included in each analysis and whether the analysis was by original assigned groups | 7 |
| Outcomes and estimation | 17a | For each primary and secondary outcome, results for each group, and the estimated effect size and its precision (such as 95% confidence interval) | 7-8 |
|  | 17b | For binary outcomes, presentation of both absolute and relative effect sizes is recommended | 7-8 |
| Ancillary analyses | 18 | Results of any other analyses performed, including subgroup analyses and adjusted analyses, distinguishing pre-specified from exploratory | 8 |
| Harms | 19 | All important harms or unintended effects in each group (for specific guidance see CONSORT for harms) | 7-8 |
| Discussion | | | |
| Limitations | 20 | Trial limitations, addressing sources of potential bias, imprecision, and, if relevant, multiplicity of analyses | 10 |
| Generalisability | 21 | Generalisability (external validity, applicability) of the trial findings | 11 |
| Interpretation | 22 | Interpretation consistent with results, balancing benefits and harms, and considering other relevant evidence | 9-11 |
| Other information | | |  |
| Registration | 23 | Registration number and name of trial registry | 2 |
| Protocol | 24 | Where the full trial protocol can be accessed, if available | The protocol could be shared on reasonable request by the corresponding author. |
| Funding | 25 | Sources of funding and other support (such as supply of drugs), role of funders | 12 |

*We strongly recommend reading this statement in conjunction with the CONSORT 2010 Explanation and Elaboration for important clarifications on all the items. If relevant, we also recommend reading CONSORT extensions for cluster randomised trials, non-inferiority and equivalence trials, non-pharmacological treatments, herbal interventions, and pragmatic trials. Additional extensions are forthcoming: for those and for up to date references relevant to this checklist, see [www.consort-statement.org](http://www.consort-statement.org).
